# New York State Veterinary Medical Society—Fourth Annual Meeting

**Published:** 1894-02

**Authors:** 


### NEW YORK STATE VETERINARY MEDICAL SOCIETY.

### PROCEEDINGS OF THE FOURTH ANNUAL MEETING.

The fourth annual meeting of the New York State Veterinary Medical Society was held at the Vanderbilt House, Syracuse, N. Y., on Tuesday and Wednesday, Jan. 9th and 10th, 1894.

#### FIRST DAY—MORNING SESSION.

The meeting was called to order at 11 A. M., President R. S. Huidekoper in the chair.

*The Chair*—The Secretary will call the roll. Roll call showed the following members present during the meeting :

Drs. J. A. Bell, W. L. Baker, Chas. Cowie, J. W. Darby, G. G. Dean, Geo. Gowland, E. D. Hayden, W. Huff, N. P. Hinkley, R. S. Huidekoper, E. B.

Ingalls, V. L. James, Wm. H. Kelley, Prof. Jas. Law, Drs. F. Morrow, C. D. Morris, E. H. Nodyne, J. A. Pendergast, M. M. Poucher, D. C. Papworth, Jno. Wende.

*Dr. Baker* moved, and seconded by Dr. Huff, that the regular business be laid over until afternoon, that the Board of Censors might hold special session to decide upon what action should be taken on the application of Dr. C. F. Moadinger and Dr. E. Pohl. Carried.

*The President*—You are all aware that the applications in question were laid over from last meeting to be acted upon at this, the annual meeting. The gentlemen who were appointed to act as Censors pro tem. at the October meeting will now report what action has been taken regarding the applications in question.

*Dr. Morris*—Regarding Dr. Pohl, I have corresponded with the President and Secretary of the Ontario Veterinary College and found he did not pass for want of knowledge of veterinary medicine. He then went to Ohio Veterinary College and was graduated from there. The standing of this college is not such as we can recommend.

*Dr. Baker*—Dr. Pohl roomed with me in Toronto; I did not think he was proficient; he did not have the ability needed.

*Dr. Cowie*—I do not think we ought to refuse admittance on account of his being unable to understand the English language.

*The President*—The Secretary will read the letters from the Ohio Veterinary College in reference to Dr. Pohl. The Secretary then read the letters as requested.

*Dr. Morris*—I will say in defence that this school has been reorganized lately. This man King is put down as an instructor; he is dean of the college according to the catalogue they send out. When he was at Toronto he would not associate with gentlemen. I do not feel that I shall retract from what I said last October. The present gentlemen are not responsible for what the other men did.

*Dr. John Wende*—I was told by a man from Ohio that the staff of this Ohio Veterinary College was partly composed of gypsies and were men who were only practical blacksmiths.

*Dr. Morris*—If these charges (if so called) were absolutely false, I would have been sued in the Supreme Court long ago. I received a letter saying that I would be if I did not retract from what I said, but so far I have not.

*Dr. John Wende*—Dr. Pohl blames me for all of this trouble regarding his graduating, and why he was plucked was one factor; the requirements are that students must practice between sessions. He presented a paper from Dr. Wm. Somerville, Jr. This certificate stated that he had practiced six months. On inquiring of Dr. Pohl about this he said he did not know about the office practice, but when Dr. Somerville had a case where people could not talk or understand the English language Dr. Pohl was asked to go as a sort of interpreter, but did not practice.

*The President*—Is he a desirable man for a member?

*Dr. Baker*—No, sir ; and I think we ought to refuse him on account of the standing of the college from which he graduated.

*The President*—Dr. Morris, was the statement in regard to the Ohio Veterinary College a statement of facts, or learned from others on hearsay?

*Dr. Morris*—It was told to me by a person who said he knew it to be so.

*Dr. Wende*—The first graduating class from that college had more members than there were members in the class during the winter term.

*The President*—A statement that they were graduating men who attended the college from six days to six weeks should not be made without definite proof. I would like to hear from Dr. Hinkley.

*Dr. Hinkley*—I have not been personally acquainted with Dr. Pohl very long only since the matter of his being accepted in the Society as a member came up, and have nothing to say against him.

*Dr. Cowie*—Dr. Morris' statements are in regard to the old college; I understand that it has been reorganized and is in better standing now.

*Dr. Baker*—I move that Dr. Pohl be rejected, and a committee of three be appointed to investigate the standing of the past and present status of the Ohio Veterinary College. We must not consider on hearsay, but must have facts. Not entertained.

*Dr. Morris*—I acted hastily in signing the application, and did not notice from what college he graduated until after I had signed it.

*Dr. Cowie*—Will Prof. Law give us his opinion on this matter?

*Prof. Law*—If the applications mentioned were accepted and questioned at the same sitting, they could be thrown out.

*The President*—I do not think we can rescind from action taken.

*Dr. Huff*—I agree with Dr. Morris. We must examine the standing of the college from which an applicant says he graduated before we accept him.

*Dr. Cowie*—I would allow that the election of Dr. Pohl to membership be allowed to stand as it is, and that he is a member of this Society, and that a committee of three be appointed to inquire into the standing of the Ohio Veterinary College.

*Dr. Dean*—I second the motion. Lost.

*Dr. Huff*—I amend to have the application laid over until the next meeting.

*Dr. John Wende*—I second the motion. Question.

*Dr. Hinkley*—Dr. Moadinger's application was received at the annual meeting in January, 1893. and examined by the Board of Censors; it had only one reference and was returned to him for the purpose of obtaining the second name as a reference, which he did and returned to me with his check for seven dollars, amount of fees and dues; I returned receipt to him.

*The President*—Dr. Pohl has been elected and is ready to make his payment, as I understand it. But Dr. Moadinger has never been elected a member.

*Dr. Huff*—I will withdraw the amendment I made to Dr. Cowie's motion.

*Dr. Morris*—I move that we adjourn until 1.30 P. M., allowing the members to get dinner, after which the officers of the Society and the Board of Censors will hold a private session with Dr. Moadinger, that he may be given an opportunity to speak in his own defence. Seconded by Dr. John Wende; carried

FIRST DAY—AFTERNOON SESSION.

Meeting convened at 1.30 P. M.

*Dr. Baker*, seconded by Dr. Poucher, that reading of the minutes of the last meeting be dispensed with, as they had been printed and circulated among the members. Carried.

*President Huidekoper* then read his annual address as follows:

GENTLEMEN:—The duty devolving upon me, as President, of having to meet you with an address of welcome is a double honor and pleasure I appreciate it the more as I see that we have gotten beyond the incubative stage, where everything rests upon the shoulders of the Association's officers, as it did in the early days of veterinary societies. Now there is a general established feeling of permanence, shown by the regular attendance of members, and by the methodical work which is done.

One year ago we had a most satisfactory meeting, here in Syracuse, although much that was proposed and discussed ventured upon new ground and was at most tentative. I was most unfortunately prevented from attending the last meeting, held in Buffalo, but was proud of the amount of work which we have recorded from those two days. Subjects of importance were so aggressively discussed that good must come from the free interchange of opinions, and the right and truth of conditions complained of must be reached. Papers of scientific value gave to all objects for reflection lasting over the days of the meeting. and personal affiliation with one's colleagues brightens one up, and makes the return to the routine drudgery of practice more cheerful.

During the last year we have added some score of new members to our roll. Our By-laws have been carefully revised to meet the requirements of our improved position and the more important functions which we are called upon to perform. The change of date for holding the annual meeting and by a longer session, including the semi annual meeting, is an excellent one, as it fixes a time when the profession is less busy and can more readily take a holiday. It was also advantageous to change the place of meeting, as we can hope by going to fresh ground to stimulate the interest of various localities throughout the State.

Charges were brought by Dr. Morris against the methods of certain schools teaching veterinary medicine. This was a bold and honest act. The United States Veterinary Medical Association has set the example for defining the standard which is required to *guarantee* a proper veterinary education. In doing so it recognized that it must necessarily keep from its doors many a well educated, reputable and desirable candidate for membership. But, the protection of its ranks for the future and the necessity of insuring proper teaching for the students of the future demanded that some decisive step be taken; and it was. While the State Association has not yet drawn its lines so rigidly, it becomes more incumbent upon individuals to scrutinize and investigate the credentials of every applicant for our ranks. This question comes before us again, and each one must argue and vote honestly upon his convictions without reference to any feeling or personal friendship, but guided only by the facts as they shall exist after the accusations and defense have been heard.

We had to blush at the condition of affairs political which warranted such a report as we received from the Committee on Legislation. The present laws in regard to veterinary practice are certainly farces, in letter and in the absurdity of attempting to execute them. A strenuous effort must be made to formulate some act which will meet the requirements of the present status of the veterinary profession. I believe, however, that more rapid good can be obtained by strict requirements in our own organization and adhesion to them by our own members. The moral effect upon the public will soon be felt.

We have sustained a sad loss during the past year in the death of Wm. Kirk, of Niagara Falls.

Another death, while not affecting our membership, has removed a prominent veterinary practitioner from the State. W. F. Carmody, M.R.C.V.S., was known by reputation, if not personally, to most of us. Dr. Carmody was a zealous, active member of the profession. He was the moving genius of the organization of a Veterinary Association in New York City a few years ago While this Association did not live a great while, its plans were excellent, as may be attested by its papers, which recently came before me, and it is to be regretted that Dr. Carmody did not have better support. Dr. Carmody was Veterinary Inspector of the National Horse

Show Association, and at its last exhibition contracted pneumonia, from which he died.

Outside of ourselves, much has been accomplished in the last year. The State Board of Health has made most satisfactory practical demonstrations of the value of tuberculin in diagnosing tuberculosis in cattle. It has further put its tests into practical use and has eradicated the disease from numerous large herds of cattle. We will have with us Dr. Cooper Curtice, of Moravia, to whom the execution of this work is to be accredited, and we shall hear of it from him.

The General Government has declared that contagious pleuro-pneumonia has been eradicated from the entire country, and promises that the freedom of our foreign cattle trade shall soon be re-established.

The United States Veterinary Medical Association held a five days' busily occupied session at Chicago. It took in hand the execution of the resolution for improvement of the status of the veterinarian of the U. S. Army, which was approved at our last meeting. It resolved to obtain a national charter, which will be a vast step towards centralizing and defining the position of the profession in its relation to the public. It took radical steps towards bringing us in closer relation with foreign veterinarians, and paved the way for the holding in the future of another International Veterinary Congress, which shall be as thoroughly so in fact as in name. The election of Dr. W. H. Hoskins, of Philadelphia, to the presidency of the United States Association is equally a personal gratification to us all, as it is an assurance of the greater work which will be accomplished in the coming year.

You will all join me in hearty thanks to and recognition of our appreciation of the work of our Secretary, Dr. N. P. Hinkley, of Buffalo, to whose labors we are indebted for the success of the Buffalo meeting and for the organization of the present meeting.

With the large amount of work before us, I will not occupy more of your time, except to give you all, in behalf of the Association, a cordial welcome and best wishes for the coming year.

Moved and seconded a vote of thanks to the President for his very able address.

*The President*—Prof. Law is with us and has a paper to read, which I know will be of great interest and benefit to us all. As he is obliged to return home by early train he would like to read it as soon as possible, thereby giving an opportunity for discussion. What is your pleasure?

*Dr. Wende*—I move we suspend regular business and listen to Prof. Law's paper. Seconded and carried.

Professor Law then presented a paper. (See page 101 this number of Journal.)

*The President*—Gentlemen, Prof. Law's paper is open for discussion.

*Dr. Hinkley* —Professor is the action of tuberculin on animals as well marked in one in the incipient stage as in one in the advanced stage?

*Prof. Law*—It is better marked in those where the tubercles are better marked or advanced. In advanced worn out cases the tubercles are more abund ant, and the tuberculin makes noticeable change. In the more advanced ones the disease is more noticeable in the membranes. A professional man could tell.

*The President*—To what degree have you found tuberculin to affect a rise in the temperature when the animal is suffering from any other disease?

*Prof. Law*—No perceptible rise except in the parturition period, which is no period to test on the cow.

*Dr. Morris*—When the tuberculin was injected, and there was no tuberculosis found, were there satisfactory results found on post-mortem examination?

*Prof. Law*—Yes, and long experienced men say it is from their wrong observations.

*Dr. Morris*—Where tubercles are attached to small membranes, is the meat as dangerous as where the kidneys, thoracic glands, or other large organs, are affected? I know in New York City meat was passed when not affecting large portions and was only on the muscles. When found on the liver the Inspector said this did not affect the meat as the secretions of the liver would overcome the trouble.

*Prof. Law* -Does this remark include the whole carcass?

*Dr. Morris*—If found in the thoracic cavity, or glands outside of the liver and kidneys, they must not use the meat.

*Prof. Law*—We have no evidence that the effect would be the same as in actinomycosis. We must leave that out in this case. Are we not bound to remove that which is endangering the public life by eating meat and milk, even when cooked?

*Dr. Morris*—The danger is in using meat and milk without being thoroughly cooked

*Prof. Law*—That is my point. Tuberculin is a chemical poison and produces a morbid process in the system by inoculation.

*Dr. Moadinger*—Does condensed milk contain the poison or is the action retained in that?

*Prof. Law*—If heat does not destroy the poison it is dangerous to use it.

*The President*—I am gratified to hear this, as it adds an important subject for discussion to the list, and one of use to all. I was called to see a herd of about fifty cows, in which it would have been impossible for anyone to have made a diagnosis of tuberculosis by the physical symptoms All of these cows had answered to the tuberculin test. Cow after cow was destroyed, and every one proved to be diseased, although in some cases the lesions were confined to a few tubercles and diseased mediastinal or mesenteric glands. I found them all to be in different stages of tuberculosis; all of them, to the observing veterinarian, would have been passed all right; by use of the tuberculin they were found to be suffering from tuberculosis.

*Dr. Cowie*—Where can tuberculin be purchased?

*Prof. Law*—Lehn & Fink, New York It does not matter about the strength; usually use four drops for a cow; use boiled water for injection with hypodermic syringe.

*Dr. James*—Does it make any difference about cows being pregnant?

*Prof. Law*—It makes no difference.

*The President*—Dr. Faust uses from one-quarter to one-half that amount.

*Dr. Baker*—In case of calcareous deposit can we get the same result?

*Prof. Law*—You will have more or less of marked rise of temperature.

*Dr. Baker*—Can you produce tuberculosis by injection of tuberculin?

*Prof. Law*—In cases where you get the genuine tuberculin you cannot, but where incipient tuberculosis exists it may be awakened and hastened by the tuberculin. Discussion closed.

Dr. Huff moved, and Dr. Morrow seconded, a vote of thanks be given Prof. Law for his very able and interesting paper. Carried.

*The President*—We will listen to the report of the Secretary.

SECRETARY'S REPORT, JAN. 9TH, 1894.

*Mr. President and Gentlemen* :—I have the honor to submit my annual report as Secretary of this Society, and it is with no little gratification that I assume this duty.

Four years ago this Society was organized and I was chosen as your Secretary, and you have seen fit at every election of officers since that time to honor me with a re-election ; a trust that I have not only felt honored and pleased with, but one that I have faithfully and earnestly tried to fulfill At every preceding report I have had encouraging and gratifying results of the conditions of the Association to submit for your consideration ; the record of the past year is no exception. and I am sure you will not contradict me when I say that veterinary science in the Empire State to-day is making such rapid and successful strides forward that our State Society ranks on an an equal with any State veterinary organization in the Union. We have good reason to congratulate ourselves on our success.

This success is not only in increased numbers of members and good financial standing, but in the interest that nearly all qualified Veterinarian Breeders' Associations and the press and public have shown in our annual and semi-annual meetings. This is largely due to the individual members who have always taken an active part, always ready to respond to any appeal for aid to support and help along the good workings of this Society.

Gentlemen, we are no longer an experiment or a theory, but a practical success. Our membership list is increasing and growing fast, numbering at present one hundred members in good standing and coming from all parts of the Empire State, from the little burgh as well as from the large and busy cities - all anxious to enroll their names on our banner.

And I am positive that not a single member of our Society to-day will say that he has not received some benefit from the pleasant and interesting meetings of this Association.

But do not stop here ; let us all continue our energies and interest until we have every qualified veterinarian in the State of New York, whose moral, social and professional standing is of such character as to allow him to be proposed to our Society and to become a member. Then and not till then can we say that we have accomplished our object and expect the reward of just recognition in the legislative body at Albany; and the municipal authority of our villages and cities, where at present butchers. cigar makers, political heelers, hoodlums, are appointed by our Health Boards as cattle inspectors and other positions which should be filled only by properly educated veterinarians.

The year of 1893 has been one long to be remembered by the veterinarians of America; the numerous successful and highly important discoveries made by our scientific workers in our laboratories · the complete stamping out of that terrible scourge pneumonia; the very important action taken and being carried out of stamping the greatest foe and destroyer of the human race (tuberculosis) by our State Board of Health, and the successful meeting at Chicago of our first Veterinary Congress held in America, where the brightest and brainiest men associated in veterinary science met and discussed important discoveries, and furnished plans for future success and prosperity, and for the public health, making it a memorable event and one never to be forgotten by those who had the good fortune to be present.

A deplorable fact to which I wish to call your attention, is the organization of numerous veterinary schools or colleges throughout the whole United States; almost every State has one or more veterinary colleges in existence; some of them, I am sorry to say, have no right to term themselves institutions for the acquirement of veterinary science. Their faculties are composed mostly of doctors whose knowledge of the veterinary art is so meagre that it is a disgrace to our profession to see their names on the public announcement sent abroad.

They give a short term of two courses from three to six months each, at the end of which time they give the fortunate, or, as I often think, unfortunate graduate a diploma and a degree which may have a good portion of the alphabet added to it composed mostly of D.V.S., M.D.V., D.V.M., V.M.D., V.S., etc.

Now while I have nothing personal against these "hurry-up colleges," I think it is the duty of every veterinarian, when an opportunity presents itself, and we all have an opportunity sometimes, to advise young men who are desirous of taking up the veterinary profession, to go to the college where he can get the most thorough education. They are all short enough as to time, but we all have hopes that the time is not far distant when our veterinary scholars—those who value their reputation—will come together and demand a better education, and at least a four years' course. I very earnestly call your attention to the urgent need of a Committee of Education and Intelligence.

During the past years our annual and semi-annual meetings have been both successful and well attended. Interesting papers were read and were well received, and no doubt brought forth good fruit.

At our last semi-annual meeting in Buffalo we were honored by the presence of Dr. W. Horace Hoskins, now President of the U. S. V. Med. Ass'n, who by his vast experience as an active member, and for years as Secretary of the U. S. V. Med. Ass'n, rendered us great and valuable aid in the important work of forming By-Laws and Constitution. At that meeting it was decided to have only one annual meetings after the present meeting, and to change the place of meeting each year to not only accommodate all members, but to try and interest all veterinarians in the different parts of the Empire State. The change, in my opinion, is a good one.

As your Secretary, during the past year, I have sent out about 3,000 invitations and have received about 200 letters, all of which have been promptly answered; I have by personal letters attempted to keep up a lively interest in the workings of the Society, but have not been able to accomplish as much as I desired owing to ill health.

The JOURNAL OF COMPARATIVE MEDICINE AND VETERINARY ARCHIVES was sent me free of charge; 250 printed reports of the proceedings of the last meeeting, held at Buffalo, Oct. 11th, 1893, and the *American Veterinary* sent 100. This very generous act, coupled with the many other favors from the journals and their editors, place the members of this Society under obligations, and I feel sure that you will appreciate it.

Before closing my report I would like to call your attention to the urgent need of forming local societies in all counties or cities where six or more veterinarians can be called together. This is one of the best methods of making our State societies more largely attended and affording better opportunities of forming social acquaintance with each other, and we will also derive very valuable and important ideas from listening to the experience and views of one another.

I am glad to see that New York City has in view the formation of such a society. As for the city of Buffalo, I shall attempt to arrange a Buffalo or Erie County society, and I hope by our next meeting to hear of several being formed throughout the State.

Our financial condition will be reported by our Treasurer, Dr. John A. Bell, and I trust that my accounts and vouchers, which are here for your inspection, will be found correct and satisfactory.

In closing my report I wish to thank most sincerely the *Veterinary Review* and the JOURNAL OF COMPARATIVE MEDICINE for their very generous act of kindness to the Society for fully publishing all of our notices and proceedings, and always ready to assist us, all free.

Also to thank the members individually and the officers for your kind assistance and consideration during the past four years, and for your kind attention to my present report, which I respectfully submit.

*Dr. Morris*—I move the report be accepted and placed on file. Seconded by Dr. Jno. Wende. Carried.

*The President*—Are there any new members present, or any applications to be acted up n? There were none presented.

*The President*—The Board of Censors will report on the applications of Drs. Pohl and Moadinger.

*Dr. Morris*—We find Dr. Pohl's election valid. and that he will be a member of this Society in good standing when his fees and dues are paid. We have decided that Dr. Moadinger has not been elected a member and desire that his application be held over until the next meeting, pending an investigation of the New York College of Veterinary Surgeons.

*Dr. Baker*—I move the report be accepted.

*Dr. John Wende*—Second the motion. Carried unanimously.

*The President*—The Committee on Legislation will report.

*Dr. Morris*—We have no report to make, as nothing has been done since the last meeting.

*Dr. Baker*—I would like to introduce an amendment to present law regarding registration.

*The President*—We will listen to it later. The Committee on By-laws and Constitution will report.

*Dr. Hinkley*—There is no report to make since last meeting. at which time the By-laws were revised. They will be printed and delivered to the members soon.

Moved and seconded the reports be accepted and placed on file. Carried.

*The President*—The Committee on Prosecution will report.

*Dr. Hinkley*—I learned of one case in Albion where an illegal practitioner was prosecuted and fined $50.00 ; in default of payment of fine he was sent to jail.

*The President*—The County Secretary's report.

*Dr. Baker*, Cortland Co.—The Local Board of Health wanted an investigation made among the dairymen and milkmen of Cortland County and the result was that several cows were destroyed on account of being affected with tuberculosis.

*Dr. Nodyne*, Wayne Co.—No report to make.

*Dr. Gowland*, Cayuga Co.—No report to make.

*Dr. Cowie*, St. Lawrence Co.—No report to make.

*Dr. Morris*, Kings Co.—On account of having left King's County soon after being appointed, I have no report to make.

*The President*—I will appoint Dr. Morris County Secretary for Duchess Co., and Dr. R. A. McLean for Kings. Others will continue to act as they stand appointed.

*Dr. Huff*, Oneida Co.—No report to make.

*Dr. Poucher*, Oneida Co.—No report to make.

*Dr. James*, Otsego Co.—Tubercnlosis seems to be prevalent in my county to quite an extent.

*Dr. Willyoung*, Orleans Co.—Although absent from the meeting, sent quite an extensive report, which was read before the meeting.

Moved and seconded the County Secretaries' reports be accepted and placed on file. Carried.

*Prof. Law*—I made a statement a year ago which appeared wrong when printed in the Journal. I stated that cases should be reported. It appeared as though I objected to having them reported. I desire this should be corrected. What I did say was:

"Of course it is in the power of veterinarians to stand upon their dignity, and to decline to report cases to the State Board of Health. But let me submit the question whether as a profession we have not a duty to fulfil to the public in this respect, and whether as citizens and veterinarians we do not owe it to humanity to make prompt report of all cases of disease likely to affect the public health?"

*The President*—I was evidently the cause for the error in the report. I said: "That unless the veterinarian was properly paid for his services, he should not feel disposed to investigate cases when the State is employing M. D.'s and non-professional men and paying them." We must look out for our own interests as well as for those of the public.

It is in order to have the Treasurer's report now, but owing to the absence of Dr. Bell, the Treasurer, the reading of the report will be postponed until to-morrow.

We will proceed to the election of officers for 1894 and 1895.

Nominations for President were made, Prof. James Law and Dr. R. S. Huidekoper.

*Prof. Law*—I must ask you to withdraw my name in favor of Dr. Huidekoper; it is impossible for me to attend to the duties of the office in connection with my duties at the University. I withdraw in favor of Dr. Huidekoper. *Dr. Baker*—I move nominations be closed. *Dr. Morris*—I second the motion. Carried.

*Dr. Morris*—I move the Secretary cast the ballot for Dr. Huidekoper as President of this Society during the time intervening between January 9th, 1894, and September, 1895. Seconded and carried.

*Dr. Hinkley*—I take pleasure in notifying you of your election as President of this Society.

Dr. Huidekoper made a few appropriate remarks, thanking the Society for the honor conferred upon him.

Nominations for Vice-President. Moved and seconded that Dr. W. L. Baker be nominated for Vice-President. Carried. Nominations closed. Moved and sconded the Secretary cast the ballot electing Dr. Baker to office. Carried. The Secretary cast the ballot and notified Dr. Baker of his election.

*Dr. Baker*—I thank you and will fill the office to the best of my ability.

Nominations for Secretary and Treasurer in order. Dr. Morris nominated for Secretary-Treasurer. *Dr. Morris*—I must decline and would move that Dr. Hinkley be re-elected to fill the office.

*Dr. Hinkley*—I must positively decline to accept the office. I have served for the past four years and think some one else ought to accept the office. I would like to see Dr. Morris or some other member of the Society elected to the office.

*Dr. Morris*—If Dr. Hinkley will accept the office I know it would be for the best interests of the Society to re-elect him.

*The President*—I sincerely hope that Dr. Hinkley will decide to continue to act as Secretary, as it is certainly for the best interests of the Society.

*Dr. Morris*—I fully sympathize with Dr. Hinkley and feel sure the members of this Society appreciate the faithful services he has rendered and hope he will continue.

*Dr. Hinkley.*—I wish you would excuse me from accepting the office. Dr. Morris is fully capable of filling the office.

Moved and seconded the nominations close. Carried. Proceed to ballot. Ballot resulted as follows:—Dr. Morris, 4, Dr. Hinkley, 10. Tellers, Dr Huff and W. B Drake. Moved and seconded that Dr Hinkley be declared elected unanimously to the office of Secretary Treasurer. Carried.

Nominations for Board of Censors in order. The following were nominated, and the vote resulted as follows, the five highest being declared elected:

| | | | |
|---|---|---|---|
| Dr. | Jno Wende | 9 | Elected |
| " | Jas. Pendergast | 6 | |
| " | V. L. James. | 5 | |
| " | W. Huff | 5 | |
| " | Chas. Cowie | 7 | Elected |
| " | E. M. Poucher | 7 | Elected |
| " | G. G. Dean | 6 | |
| " | Jno. A. Bell | 10 | Elected |
| " | C. D. Morris | 9 | Elected |

The five gentlemen receiving the largest number of votes were declared elected for the ensuing year. Drs. Jno. Wende, Charles Cowie, M. M. Poucher, Jno. A. Bell, C. D. Morris.

*The President.*—It is provided in the by-laws that one more member be elected to act with the Committee on Legislation. The following were nominated: Dr. C. D. Morris, Prof. Jas. Law. The vote showed the following result:

Dr. Baker 0, Dr. Morris 2, Prof. Jas. Law 11. Prof. Law declared elected to act on Committee of Legislation.

*The President.*—We will listen to the reading of Dr. Morris's paper: Subject, Professional Etiquette. See page 109 this journal.

*The President.*—Gentleman, you have heard the paper read by Dr. Morris. What is your pleasure?

*Dr. Baker.*—I move a vote of thanks to Dr. Morris for his very able paper.

*Dr. Hinkley.*—I second the motion and will have the paper published. Carried.

*Dr. Morris.*—I move we go into executive session. Dr. Huff seconded the motion. Carried.

*Dr. Moadinger.*—I would like to make a few remarks, Mr. President.

*The President.*—Please make your remarks in writing and send them in to the Secretary.

*Dr. Moadinger.*—I will do so. (Dr. Moadinger then withdrew from the room.)

*Dr. Morris.*—Will Dr. Baker submit the amendment to the present law?

*The President.*—Dr. Baker will please submit the amendment.

Dr. Baker read the law as existing at present in the State of New York, and adds an amendment that the fine of $50 bc divided equally between the County Treasury and the person laying the information before the Court.

*Dr. Wende.*—I think it unconstitutional.

*Dr. Morris.*—On what grounds?

*Dr. Wende.*—Because it comes in competition with the law in existence now.

*Dr. Baker.*—I do not think so. This simply changes the division of fine. Discussion continued a short time.

*Dr. Morris.*—I move we adjourn until 7.30 P. M. Seconded by Dr. Huff, and carried.

EVENING SESSION, JANUARY 9th, '94.

Meeting called to order 7.30 P. M. The President: We will listen to a few remarks from Dr. Moadinger regarding his application.

*Dr. Moadinger* related the circumstances connected with making his application and obtaining references, sending check for amount of fees and dues and presented receipt for same. Dr. Moadinger's statement corroborated that of Secretary Hinkley, and in closing he asked to withdraw his application for membership. His request was placed in the form of a motion. Seconded, voted on, and carried unanimously.

*The Secretary* then paid Dr. Moadinger the $7 which he had received on Jan. 17th, 1894, for fees and dues, and in return took back the receipt held by Dr. Moadinger.

A discussion was entered into and carried to quite an extent regarding the prosecution of men who were practicing veterinary surgery without duly registering.

*Dr. Baker* moved that the Prosecuting Committee be continued as they were.

*The President* added the following as an amendment: "And empowered to act entailing such expenses as will meet the approval of the Secretary-Treasurer in prosecuting any cases that may come under their observation and those who are practicing illegally." Dr. Baker's motion as amended seconded by Dr. Cowie. Carried.

*Dr. Baker.*—I move that a committee of three be appointed "of which the President shall be one" to investigate the standing of the Ohio Veterinary College, and the College of Veterinary Surgeons of New York. Seconded by Dr. Bell Carried.

The subject of de horning cattle was brought before the meeting and discussed

*The President.* —I believe it is good practice to sear the wound after sawing off the horn.

*Dr. Morris* cites cases where the farmers in his community use caustic potash on young calves to take off the young horn and stop the growth of same. A short discussion of the subject followed.

*Dr Morris.*—I would like to hear from some of the members in regard to treating old sores where they continued to form scabs and false growth. Cites a case he had charge of.

*Dr. Bell.*—I use tannin with good success.

*Dr Hinkley.*—I think you will find Dr. Bell's mode of treatment together with compresses very good in any of those cases.

*Dr. Morris.*—How do you treat parturiant apoplexy?

*Dr. Cowie.*—I have no special treatment. I use hot and cold water alternately with blankets, with good success. I use it from six to eight hours after birth of calf. I have treated four or five cases in that manner.

*Dr. Baker.*—I have good success with hypodermic injections of strychnia.

*Dr. Morris.*—I tried large doses of ammonia, strychnia and cold water as recommended by Dr. Sutterby, but was not very successful in my treatment.

*Dr. Cowie.*—I do not think medicine given internally has any effect when the animal is in a comatose conditon.

*Dr. Morris.*—I have given purgative when in a comatose condition and had good results from it.

*Dr. John Wende.*—I have had good success by using aromatic spirits of ammonia and whiskey.

*Dr. Bell.*—I know cases where good results have been obtained from the use of aromatic spirits of ammonia, turpentine and linseed oil given alternately in doses of two ounces each. I also know of recovery from the use of aconite and belladonna.

*Dr. Morrow.*—I know of good results being obtained from the use of aconite and belladonna in medium doses every hour. At the same time we cannot depend upon medicine as we can upon surgery.

*Dr. Hinkley.*—That depends upon the class of medicine you buy. Mentions esorine, pilocapine, and the good results obtained from their use.

*Dr. Morris* then offered the following resolutions :

RESOLUTIONS.

*Whereas*, the Secretary of Agriculture in his annual report desires to reform the present system in relation to appointing veterinary inspectors and assistant inspectors in the Bureau of Animal Industry, asked that, "as a condition preceding all such appointments the applicants shall exhibit to the United States Civil Service Commission his diploma from an established, regular, and reputable veterinary college, and that this be supplemented by such an examination in veterinary science as the Commission may prescribe."

*Resolved*, that this Society unanimously favors this portion of the Secretary's report; that the Society desires to co-operate with the Hon. Secretary toward a realization of this high ideal and much needed reform in the Bureau.

*Dr Morris.*—I think that parties ought to be appointed after Civil Service examination, and am very much pleased to see the Hon. Secretary of Agriculture taking this course, and I think the veterinarians ought to assist the Secretary in this matter all they possibly can. I offer this resolution as coming from the Society as an approval of the act of the Secretary.

*Dr Huff.*—I move the report be accepted, and the Treasurer, John A. Bell be released from his bonds.

*Dr. Baker.*—Second the motion. Carried.

*Dr. Bell.*—I move the Secretary make a list of all members who are in arrears for fees, dues and assessments and read the same at the afternoon session.

*Dr. Gowland.*—I second the motion.

*The President.*—I will read the names of the members whom I have appointed to act on the different committees during the ensuing year.

*The President.*—In regard to the duties of the Prosecuting Committee, I would advise them to consult among themselves regarding the test case at Albion, and get a copy of the case and send same to any who may want to make prosecution in any part of the State ; also consult with the Secretary-Treasurer and abide by his descision as to the amount of expense to be entailed in making said prosecutions.

*Dr. Bell.*—I would advise having copies of the case made and sent to all members of this Society.

*Dr. Jno. Wende.*—We ought to take some action in regard to the institutions that are issuing diplomas for practicing veterinary dentistry. There are one or two such in Toronto, Ont. One man named Lucas, who is a carpenter by trade, has a certificate of some corporation and uses it in place of a veterinarian's diploma. He has been known to issue certificates to young men leaving Canada, giving them the right to practice veterinary dentistry. There is one Dr. McPherson in Canada who issues certificates for the same purpose.

*Dr. Baker.*—I have seen the certificates issued by Dr. McPherson, and know that he issues them. I believe veterinary dentistry ought to come under the head of veterinary surgery. If a veterinarian wants to make a specialty of dentistry, let him do so after graduating from a veterinary college or university of reputable standing.

*The President.*—I agree with Dr. Baker, and would like to ask him if he learned anything from Dr. McPherson or any of his pupils that he did not learn at college?

*Dr. Baker.*—No, sir.

*Dr. Cowie.*—Do any of you know personally of Dr. McPherson having issued certificates or diplomas?

*Dr. Jno. Wende.*—I have seen two certificates in Buffalo, one issued by McPherson and one issued by Lucas, both parties holding them were practicing veterinary dentistry in Buffalo.

*Dr. Jno. Wende.*—I move to appoint a committee of three to draw up a resolution condemning the institute issuing dental diplomas.

*The President.*—I would amend that to include all institutions issuing diplomas for practice of veterinary dentistry only, as that is considered a part of veterinary surgery, and no one ought to be allowed to practice it unless a graduate in veterinary medicine and surgery.

*Dr. Hinkley.*—I agree with Dr. Huidekoper. Anyone who has graduated from a veterinary college ought to have learned enough about dentistry to be able to practice it as met with. I think we ought to condemn the schools issuing the certificates or diplomas as has been stated by the members here.

*Dr. Cowie.*—Do you intend to condemn the man who, after graduating, takes a special course in dentistry?

*The President.*—No, sir; not those who graduate, but the institution that teaches veterinary dentistry and does not teach veterinary surgery.

*Dr. Baker.*—I second Dr. Wende's motion as amended by Dr. Huidekoper. Carried.

*The President.*—I will appoint Dr. Jno. Wende, Dr. W. L. Baker, Dr. E. H. Nodyne a committee of three to draw up the resolution.

RESOLUTION.

WHEREAS, Certain persons giving instruction in veterinary dentistry issue certificates in the form of diplomas, and

WHEREAS, We do not recognize veterinary dentistry as a special and separate art or science, but only as an item in the education and practice of every qualified veterinarian,

*Resolved*, That we deprecate the assumption of the title of veterinary dentist as arrogant and misleading to the public; be it further

*Resolved,* That we condemn the issuing of certificates of proficiency in veterinary dentistry which approach the form of diplomas and are intended to mislead the public, and that we do not recognize such certificates as entitling the bearer to assume the title of veterinary surgeon or register as a practitioner of veterinary medicine.

JNO. WENDE, V.S.,
W. L. BAKER, V.S.,
E. H. NODYNE, V.S ,
Committee.

The following section was discussed but not included in the resolution:

*Resolved,* That where such so-called veterinary dentists are called in to attend to horses' mouths in the stables in which we practice, or where we are called on to examine in any way the mouths of horses which have been treated by the so-called specialists, that we advise charging the owner consultation fees in place of ordinary visit fees.

*Dr. Jno. A. Bell* read a communication on practical experience of his regarding nail in the foot. See page 119 this Journal.

This paper was read for the purpose of getting the views of different members regarding the treatment of wounds of the foot with hot and cold water, or hot and cold applications.

*Dr. Bell.*—I would like to ask Dr. Wende why he uses cold applications in laminitis and warm water in other cases.

*Dr. Wende.*—I judge from the results I get. I treat different cases in different manner as the symptoms call for.

*Dr. Baker.*—I favor cold water. Heat favors exudation which I do not like. I have used ice bags, applying them from the hoof to the body, with very good results. I used warm water for two years in my practice and the results obtained were very unsatisfactory.

*Dr. James.*—I use warm applications. I believe them to be more agreeable to the patient.

*Dr. Huff.*—In acute form I use hot applications and cold applications later with very good results.

*Dr. Jno. Wende.*—I use three to four ounces of fld. ext. of jaborandi in one pint of water, blanket the horse and let him stand until after the action of the jaborandi and allow him to stand in cold water. He is generally cured in from 24 to 48 hours.

*Dr. Hinkley.*—I find we must treat different cases differently. In some cases of laminitis, following indigestion, I find hot water with aromatic spirits of ammonia give very satisfactory results.

*Dr. Jno. Wende.*—I would not use jaborandi where the pulse is over 60 degs.

*Dr. Bell.*—Where a nail strikes the foot I like cold applications best.

*Dr. Wende.*—I use hot water in those cases to prevent tetanus.

*Dr. Papworth.*—Will using hot poultices be more liable to cause tetanus than hot water?

*Dr. Wende.*—I think so.

*The President.*—I use both hot and cold water as circumstances admit regarding facilities for taking care of the case. I sometimes poultice the whole length of the leg. I do not think there is danger of tetanus where the knife is freely used and the wound is thoroughly opened, but believe there is danger of it when the wound is kept closed.

I do not think the pathology of laminitis is the same in all cases. Where it is caused by over-eating it is different from a case caused by influenza or other diseases of that nature My treatment in the first-mentioned cases is to bleed from the jugular vein to the extent of four or five quarts, and in other cases pare the foot very thin and bleed from the toe ; cut away the wall and get a free hæmorrhage. I have used nitrate of potash with good results.

*Dr. Dean* --Dr Wende, would you advise using cathartics in laminitis?

*Dr. Wende.*--Not in all cases.

*Dr. Hinkley.*--I agree with Dr. Huidekoper in the treatment of the wounds. Keep them open, and I treat with peroxide of hydrogen with very good results.

*Dr. Jno. Wende.*—I have good results by cutting away the sole all that I can and treat the wound antiseptically.

*Dr. Huff.*--I treated a case which I turned away apparently well. After horse was in use a few days I was sent for and found him suffering from tetanus. Made good recovery.

*The President*—Dr. Huff, in your case I think if the horse had died you would have found a cell of pus up in the foot on post-mortem examination. I find it a good idea to cut away all walls covering inflammatory products in foot trouble or chronic founder

*Dr. Dean.*--How do you find inflammatory points?

*The President*—By feverish inflammatory condition and soreness

*Dr. Cowie.*—Do you not have trouble with granulation afterward?

*The President*—Not with proper dressing.

*Dr. Cowie.*--I mean from acute inflammation from puncture?

*The President.*—No, sir; I only cut the ends of the papilla until drops of blood come through from pressure.

*Dr. Cowie.*—I move a vote of thanks to Dr. Bell for his very able and interesting paper.

*Dr Baker.*--I second the motion. Carried. Adjourned for dinner.

WEDNESDAY, JAN. 10TH, 1894.—AFTERNOON SESSION.

Meeting called to order at 2 P. M

*The President.*—The Secretary will read any communications that will be of interest to the members. Communications were read from Drs. Minchin, Dutcher and Austin.

A discussion regarding the place of the next meeting followed, after which a motion was made by Dr Ingalls, seconded by Dr. Huff, that a ballot be taken to decide the place of next meeting. Carried The ballot showed that the majority favored going to Albany. So decided.

*The President.*—Will the Secretary please read the names of the delinquent members, and I would suggest the Secretary write each delinquent member that his name has been brought before the members of this meeting and that we must ask him to pay up all indebtedness within ninety days, or he is liable to suspension at the next meeting.

*Dr. John A. Bell.*—I would offer this as a resolution :

That the Secretary write to all delinquent members that their names are before the members of the Society on account of non-payment of fees, dues and assessments, and that ninety days from date of first notice a second notice be sent, and report results at the next meeting.

*Dr. Baker.*—I move the resolution be accepted and passed.

*Dr. Huff.*—I second the motion. Carried.

*The President.*—We are simply taking a course similar to that of the United States Veterinary Medical Association, and we must insist upon having these small accounts promptly paid, and if they do not intend to pay, we want them to say so.

*Dr. Hinkley.*—I would make a motion that after this date, where an assessment is made, that applicants who are elected to membership at the same meeting shall be exempt from paying that assessment.

*Dr. Poucher.*—I second the motion. Carried.

*The President.*—We will listen to the reading of Dr. Willyoung's paper on "Comparative Ovariotomy" Owing to the absence of Dr. Willyoung, the Secretary's assistant, Mr. Drake, will read the paper.

Comparative Ovariotomy will be published March Journal.

*Dr. Bell*—I approve of Dr. Willyoung's method of operating. It is nearly the same as I follow.

*Dr. Wende.*—I have operated on some; usually remove to the forks of the horns.

*The President.*—I do not use anæsthetics in operating on those cases. I think it is better without them.

*Dr. Gowland.*—I agree with Dr. Huidekoper.

*The President.*—I have been asked what dressing I use in old and indolent sores, shoe boils, etc. Would say I find a mixture of Bals. Peru. 1 pt., Iodoform 1 oz., very good to use in all cases of granulating surfaces, especially shoe boils.

*Dr. Huff*—At what stage do you use the above?

*The President.*—After removing the fibrous tumor.

*Dr Hinkley*—In dissecting a fibrous mass, do you take out all small fibres?

*The President.*—Yes, I do.

Discussion closes.

*Dr Hinkley.*—Inasmuch as some of our members notified me that they would read a paper at this meeting, and I have published it together with the subjects given me by them, and that members have come here expecting to hear these papers read and discussed, and to take part therein, and as the essayists have failed to do as they promised and have given no reason for their absence, I would offer the following resolution:

WHEREAS, Inasmuch as certain members of our Society have notified and promised the Secretary of this Society that they would write, read and defend certain essays on their chosen subjects at this annual meeting to be held at Syracuse, N. Y., Jan. 9th and 10th, 1894;

WHEREAS, The failure of said members to either attend, send papers, or excuses to the Secretary, stating why they could not attend the meeting, and by such flagrant neglect have caused delay and annoyance to the attending members of the Society; be it

*Resolved*, That members having essays to read be earnestly requested either to be present to read and defend their papers or send said papers, or a written notice of their inability to do so, and not subject the members of the Society to the above-mentioned annoyance and delay.

*Dr. Bell*—I move Dr. Hinkley's resolution be accepted and placed on the minutes of the proceedings.

*Dr. Cowie.*—I second the motion. Carried.

*The President.*—Will Dr. Wende please read the paper which he has?

*Dr. Wende.*—I did not expect to read a paper at this meeting, but owing to the absence of essayists, will do as the President requests.

Dr. Wende's paper on Pyo-septhamie, see page 116 this Journal.

*Dr. Jno. A. Bell.*—We meet with very few cases in this country.

*Dr. Wende.* —I had one case; a colt of my own which I lost with it.

*The President.*—Dr. Wende, have you had many cases?

*Dr. Wende.*—Quite a few.

*Dr. Bell.* Where did you do the cutting?

*Dr. Wende.*—I cut the umbilicus lengthwise open to the body to find the vein. I put in human catheter and use sol creoline with bulb syringe.

*Dr Huff.*—How old is the colt, usually?

*Dr. Wende.*—From three days to six weeks.

*Dr. Huff.*—Do you find any germs?

*Dr Wende.*—No, sir.

*Dr. Bell.*—I thought it was caused by a germ.

*Dr. Wende.*—No, sir; it is caused by absorption.

Discussion closed.

*The President.*—I will appoint the following gentlemen as essayists for the next meeting: Dr. W. L. Baker, Dr. Jno. Wende, Dr. Jno. A. Bell, Dr. Wm. H. Kelley, Dr. W. Huff.

*Dr. Hinkley.*—Before we adjourn I wish to offer a resolution of thanks to the members of this Society who have furnished and read the interesting and very able papers at this meeting; also the members for their attention and interest displayed during the sessions, and to the proprietors of the Vanderbilt House for their kind and courteous hospitality.

*Dr Wende.*—I move that the resolution be passed as an expression of the members.

*Dr. Baker.*—I second the motion. Carried.

The meeting adjourned, subject to the call of the Secretary.

The next meeting will be held at Albany during the month of September, 1894.

RUSH S. HUIDEKOPER, *President.* NELSON P. HINKLEY, *Secretary.*

---

### APPENDIX.

### OFFICERS 1894–5.

| | | |
|---|---|---|
| President | Rush S. Huidekoper | 155 West 56th Street, N. Y. |
| Vice-President | W. L. Baker | Cortland. |
| Secretary-Treasurer | N. P. Hinkley | 395 Ellicott Street, Buffalo. |

### CENSORS.

| | |
|---|---|
| John A. Bell | Watertown. |
| John Wende | Buffalo. |
| C. D. Morris | Pawlings. |
| Charles Cowie | Ogdensburg. |
| E. M. Poucher | Oswego. |

### COMMITTEES 1894.

*Committee of Arrangements*—The Secretary-Treasurer (by By-laws), Dr. W. H. Kelley, 295 West Avenue, Albany; Dr. Thomas Giffen, New York.

*Committee on Legislation*—The President, the Secretary-Treasurer (by By-laws), Prof. James Law.

*Committee on By-Laws*—The Secretary-Treasurer (by By-laws), C. D. Morris, John A. Bell.

*Committee on Investigating Colleges*—The President (by resolution), C. D. Morris, W. L. Baker.

*Auditing Committee*—J. W. Darby, Dr. Pendergast, Dr. Dean.

*Prosecuting Committee*—W. L. Baker, John A. Bell, John Wende.

